# Effect of the glucagon-like peptide-1 (GLP-1) receptor agonist semaglutide on alcohol consumption in alcohol-preferring male vervet monkeys

**DOI:** 10.1007/s00213-024-06637-2

**Published:** 2024-06-17

**Authors:** Anders Fink-Jensen, Gitta Wörtwein, Mette Kruse Klausen, Jens Juul Holst, Bolette Hartmann, Morgan Thomsen, Maurice Ptito, Amy Beierschmitt, Roberta M. Palmour

**Affiliations:** 1 Laboratory of Neuropsychiatry, Mental Health Centre Copenhagen, Mental Health Services in the Capital Region of Denmark, University of Copenhagen, Hovedvejen 17, Frederiksberg, DK-2000 Denmark; 2 Department of Clinical Medicine, Faculty of Health and Medical Sciences, University of Copenhagen, Copenhagen, Denmark; 3 Department of Public Health, Faculty of Health and Medical Sciences, University of Copenhagen, Copenhagen, Denmark; 4 Novo Nordisk Foundation Center for Basic Metabolic Research, Department of Biomedical Sciences, University of Copenhagen, Copenhagen, Denmark; 5 Department of Neuroscience, Faculty of Health and Medical Sciences, University of Copenhagen, Copenhagen, Denmark; 6 School of Optometry, Université de Montréal, Montréal (Qc), Canada; 7 Behavioural Science Foundation, St. Kitts, Saint Kitts and Nevis; 8 Faculty of Medicine and Health Sciences, McGill University, Montréal (Qc), Canada

**Keywords:** Semaglutide, GLP-1, Alcohol, Alcohol use disorder, AUD, Monkey, Vervet, Non-human primates, Self administration

## Abstract

**Rationale:**

Glucagon-like peptide-1 (GLP-1) receptor agonists reduce alcohol consumption in rodents and non-human primates. Semaglutide is a new long-acting GLP-1 receptor agonist, widely used in the clinic against type 2 diabetes and obesity. It is also reported to reduce alcohol intake in rodents.

**Objectives:**

This study investigates the possible inhibitory effect of semaglutide on alcohol intake in alcohol-preferring African green monkeys.

**Methods:**

We performed a vehicle-controlled study on male monkeys that had demonstrated a preference for alcohol. In the monkeys selected for voluntary alcohol drinking, alcohol consumption was measured for ten days at baseline (Monday to Friday for two weeks). During this period, the monkeys had access to alcohol 4 h per day and free access to water 24 h per day. After two weeks of baseline measurements, the monkeys were randomized to semaglutide or vehicle. Each group consisted of ten monkeys, and the two groups were balanced with respect to baseline alcohol intake. Following the baseline period, the monkeys were treated with escalating doses of semaglutide (up to 0.05 mg/kg) or vehicle subcutaneously twice weekly for two weeks during which period alcohol was not available. After uptitration, the monkeys had access to alcohol 4 h daily for 20 days (Monday to Friday for 4 weeks), and alcohol consumption was measured. During this alcohol exposure period, treatment with semaglutide (0.05 mg/kg twice weekly) or vehicle continued for three weeks followed by a one-week washout period.

**Results:**

Compared to the vehicle, semaglutide significantly reduced alcohol intake. There were no signs of emetic events or changes in water intake.

**Conclusions:**

These data demonstrate for the first time the potent effect of semaglutide in reducing voluntary alcohol intake in non-human primates and further substantiate the need for clinical trials investigating the effect of semaglutide in patients with alcohol-use disorder.

## Introduction

Harmful use of alcohol causes three million deaths yearly (WHO. Global Status Report on Alcohol and Health 2018), and alcohol use disorder (AUD) is a disabling condition associated with increased morbidity and mortality, and decreased quality of life (Carvalho et al. 2019). A cumulative all-cause 15-year mortality risk of 29% after a first-time hospital admission due to an alcohol-related problem has recently been reported in Denmark (Askgaard et al. 2020). Treatment of AUD with cognitive-behavioural therapy is well documented, but clinical guidelines recommend the add-on of pharmacological therapy in moderate to severe AUD. However, pharmacological therapy has not gained widespread acceptance. Consequently, there is an urgent need for novel molecular targets for the medical treatment of AUD (Swift and Aston 2015) and a better understanding of the pathophysiological and psychological underlying mechanisms.

Glucagon-like peptide-1 (GLP-1) is a peptide produced by intestinal endocrine L-cells (Holst 2007) as well as by neurons of the nucleus tractus solitarius (NTS) (Göke et al. 1995). GLP-1 is released in the intestinal tract upon food intake, regulates glucose homeostasis, and reduces appetite through peripheral and central mechanisms of action (Holst 2007). GLP-1 receptor agonists (GLP-1RAs) have been reported to reduce self-administration of cocaine, amphetamine, nicotine, opioids, and alcohol in rodents (Eren-Yazicioglu et al. 2021; Klausen et al. 2022a, b) as well as alcohol intake in monkeys (Thomsen et al. 2019, 2020).

In humans, several clinical studies have investigated the effects of GLP-1 RAs against nicotine consumption. The GLP-1 RA exenatide adjunct to nicotine patch was reported to facilitate smoking cessation (Yammine et al. 2021), whereas no effects on smoking cessation were observed when the GLP-1 RA dulaglutide was tested (Lengsfeld et al. 2023; Lüthi et al. 2024). Very recently, a retrospective cohort study has reported on an association of semaglutide with reduced incidence and relapse of cannabis use disorder in real-world populations (Wang et al. 2024).

Currently, several registered clinical trials are ongoing to evaluate the effect of semaglutide on alcohol consumption (ClinicalTrials.gov identifier; NCT06015893; NCT05891587; NCT05520775; NCT05892432; NCT05895643), and on smoking cessation (ClinicalTrials.gov identifier: NCT05610800; NCT05530577).

We previously emphasized the need for conducting clinical trials that are randomized and placebo-controlled, aimed at investigating the impact of GLP-1 RAs in patients diagnosed with AUD (Fink-Jensen and Vilsbøll 2016). Recently such a trial was completed (Klausen et al. 2022a, b). Both the exenatide and placebo groups showed a 50% reduction in alcohol consumption and heavy drinking days, with no statistically significant difference between the two groups. However, when investigating patients with a Body Mass Index (BMI) above 30, alcohol consumption and number of heavy drinking days were lower in the exenatide group compared to the placebo group. In addition, alcohol cue-induced activation in reward-related brain areas was reduced in the exenatide group compared to the placebo group (Klausen et al. 2022a, b). Further highlighting a potential role for GLP-1RAs in AUD management, a recent cohort study suggested that using GLP-1 analogs prescribed for their currently approved indications was associated with a lower incidence of alcohol-related events (Wium-Andersen et al. 2022).

In previous studies, we examined the impact of GLP-1RAs, specifically exenatide and liraglutide, in alcohol-preferring vervet monkeys. Our findings demonstrated a substantial reduction in alcohol consumption, ranging from 25 to 50% (Thomsen et al. 2019, 2020). The newer and more potent GLP-1RA semaglutide is used in the clinic to treat type 2 diabetes (Andreadis et al. 2018) and obesity (Wilding et al. 2021). The acute effect of semaglutide on alcohol consumption has recently been investigated in rodents, where it potently decreased alcohol intake (Aranäs et al. 2023; Chuong et al. 2023), but to the best of our knowledge, data on the effect of semaglutide on alcohol consumption in non-human primates have not yet been reported. Therefore, our goal was to investigate the effect of semaglutide on alcohol consumption in alcohol preferring vervet monkeys, and we hypothesized that semaglutide would potently decrease the overall alcohol intake.

## Materials and methods

### Subjects and housing

Young adult male vervet monkeys (*Cercopithecus aethiops*) weighing 4.4–6.5 kg were assigned to either a dose-finding experiment (3 animals) or a vehicle-controlled experiment (20 animals). Here, alcohol preference was defined as drinking at least 3 g alcohol/kg body weight/4 hours. All the animals were colony-born at the Behavioural Science Foundation (BSF), St Kitts, and the experiment was conducted at this site. The monkeys were drug and experimentally naïve, except for prior screening for alcohol preference. The animals were socially housed in outdoor wire cages 2 × 3 × 2 m as previously described (Palmour et al. 1998) and were provisioned with High Protein Monkey Diet from Harland Teklad (Madison WI, USA) supplemented with fresh local fruit; water was available *ad libitum* both in normal housing and during the experiment. To record the individual drinking pattern, the monkeys were removed from the social housing and individually housed in single cages during which time they always had visual and olfactory communication with one another. The pilot study and the main study took place during the months of April-June 2020, when the typical ambient temperature was 23–25 °C. The study was approved by the Behavioural Science Foundation Institutional Animal Care and Use Committee (BSF IACUC), operating under the auspices of the Canadian Council on Animal Care (Canadian Council on Animal Care Good Animal Practice registration A5028) (approval number BSF 2001). The procedures used in the present study were all covered by standard operating procedures (SOP) approved by the BSF IACUC.

## Experimental groups and design

### Semaglutide pilot study

To find a tolerable dose for the main experiment, a pilot study was performed on three monkeys each weighing around 5 kg placed in single cages during the pilot experiment. We had previously tested the GLP-1 receptor agonist liraglutide in alcohol preferring monkeys at a *daily* dose of 0.05 mg/kg subcutaneously (s.c.) (Thomsen et al. 2019). In patients, liraglutide is administered *daily* with a target dose of 1.8 mg s.c. or 3.0 mg s.c. depending on the indication (type two diabetes or overweight/obesity, respectively). Semaglutide, in contrast, is administered to patients *weekly* with a target dose of 1.0 mg s.c. or 2.4 mg s.c. depending on the indication (type two diabetes or overweight/obesity, respectively). Based on this information, we started with a 0.01 mg/kg dose administered s.c. *twice weekly* and observing for possible side effects, e.g., emetic events or reduced food intake. In case of side effects, the dose was decreased stepwise to a tolerable dose (i.e., no profound side effects observed), or increased stepwise up to max 0.05 mg/kg s.c. *twice weekly.* For subcutaneous injections of semaglutide or vehicle, the monkeys were immobilized in their cages according to Standard Operating Procedure (SOP) 10.2, twice weekly. Following injections, the monkeys were observed for four hours on an hourly basis for side effects including emetic events and changes in food intake. The titration plan was as follows: If the initial dose (0.01 mg/kg) was tolerated, i.e., no profound observed side effects, a dose of 0.03 mg/kg s.c. *twice weekly* was used. If this dose was also tolerable, a dose of 0.05 mg/kg s.c. *twice weekly* was investigated – and if tolerable, this dose was used in the main study. None of the monkeys in the pilot study were used in the main experiment. Dosing twice weekly was based on the dosing regimen in humans (once weekly) and the generally faster metabolism in non-human primates compared to humans.

### Semaglutide main study

**Fig. 1:**
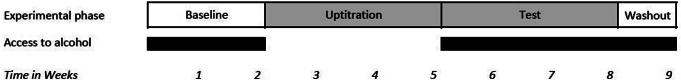
Schematic representation of the experimental phases of the semaglutide main study. Open boxes represent no treatment, grey boxes represent twice weekly administration of semaglutide or vehicle. For access to alcohol, black bars represent daily 4 h access, no bars represent no access

Baseline alcohol consumption was evaluated for the 20 monkeys chosen for the main study using the standard scheduled access paradigm previously described (Palmour et al. 1998). The animals had free access to 10% (w/v) alcohol solution as well as water (two bottle-choice) 4 h/day from 9 a.m. to 1 p.m. 5 days/week (Monday to Friday). Baseline alcohol intake was measured for 2 weeks (Monday to Friday), whereafter the animals were allocated to the semaglutide group or vehicle group balanced both for alcohol intake and body weight. After these two weeks, animals were returned to their social group housing for three weeks of semaglutide/vehicle treatment. The monkeys were identified by shaving marks enabling the researchers to differentiate between semaglutide- and vehicle-treated monkeys. Semaglutide or vehicle (saline solution) was injected s.c. using escalating dosing, as follows: 0.01 mg/kg semaglutide twice weekly for week one, 0.03 mg/kg semaglutide twice weekly for week two and 0.05 mg/kg semaglutide twice weekly for week three (as shown in Fig. 1). Alcohol was not available during this up-titration procedure. No adverse effects were observed during this period. Following the three weeks of up-titration, animals were returned to individual caging, access to alcohol was re-introduced and drinking pattern was documented for the following four weeks (Monday to Friday), in which semaglutide was still administered at a dose of 0.05 mg/kg s.c. twice weekly (Tuesday and Friday) for three weeks. Alcohol and water intake monitoring continued for one week of wash out, during which semaglutide or vehicle were not administered.

Both the volumes of water intake and alcohol intake were measured by technicians who were blind to treatment. Blood samples were collected for plasma semaglutide quantification by femoral venipuncture (BSF SOP 4.1) during the last week of drug treatment. Body weights were recorded at the start and at the end of the experiment.

### Plasma semaglutide analysis

Semaglutide concentrations were measured with a radioimmunoassay originally described by Deacon et al.(Deacon et al. 2002). Antibody (code no. 98,302) specific for the intact N-terminus of GLP-1 was used and semaglutide served as the standard. This plasma assay will besides semaglutide also measure intact biologically active GLP-1 (that is, GLP-1(7–36)NH2 and GLP-1(7–37) which under normal circumstances are present in low picomolar concentrations. Plasma was diluted x100 with assay buffer before analysis. The measurable range was 80-5120 pM.

### Pharmacological agents

Semaglutide (Ozempic®) injection pens (Novo Nordisk) were purchased from the Rigshospitalet Hospital Pharmacy. The semaglutide was further diluted in a saline solution and a physiological saline solution was used as vehicle control. The alcohol used in the present experiment was local rum produced in St. Lucia (Denrose Strong Rum, 80% alcohol *v/v*) and diluted in water to a final ethanol concentration of 10% (*w/v*).

### Statistical analyses

Alcohol intake and water intake were analyzed by an ANOVA with treatment group as between-subjects factor and week as repeated-measures factor. Šídák’s multiple comparisons test was used for post-hoc comparisons. All data are presented as means ± standard error of the mean (S.E.M.). Effects showing *p* < 0.05 were considered significant. Analyses were performed using GraphPad Prism (version 10.1.2).

## Results

### Semaglutide pilot study

No emetic events or other signs of nausea or skin irritation were detected at any of the doses tested. Water and food intake were not affected.

### Semaglutide main study

**Fig. 2:**
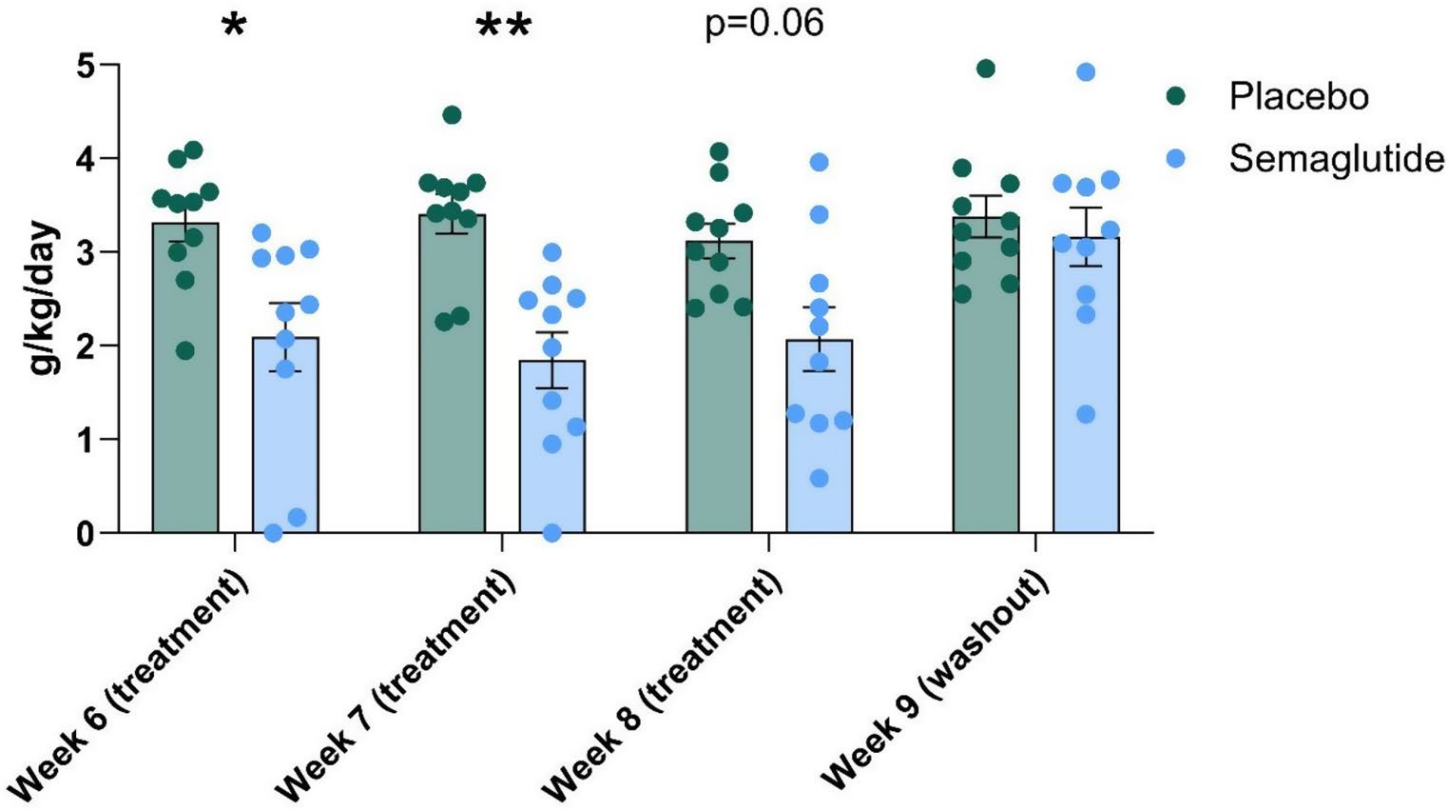
Average daily alcohol intake (g/kg/4 hours) at week 6, week 7, week 8 and week 9 (washout) (Monday to Friday). Group size = 10. **P < 0.01, *P < 0.05, semaglutide vs. vehicle, Šídák’s multiple comparisons test. Data are presented as mean ± S.E.M and points represent individual values

**Fig. 3:**
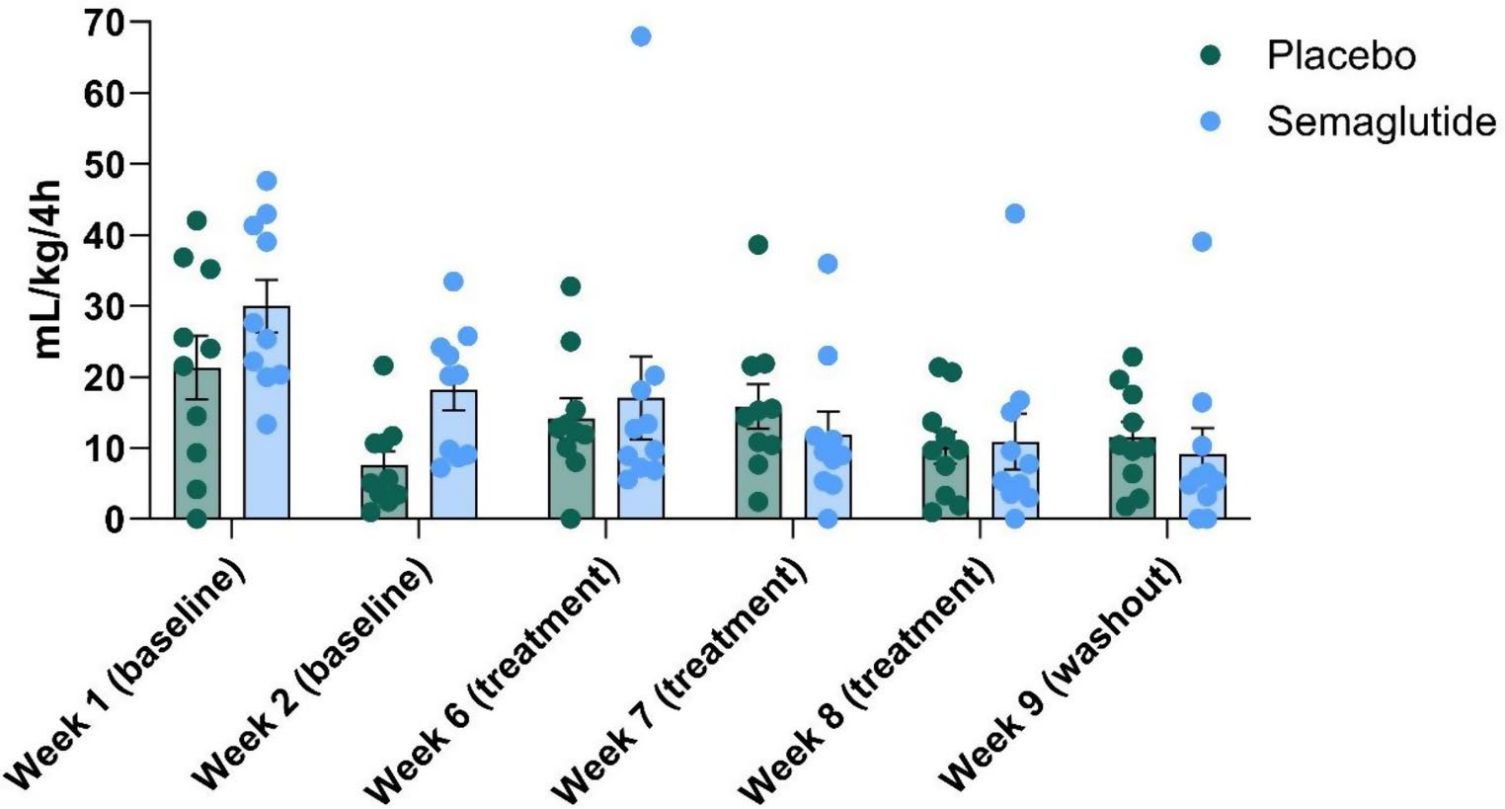
Average daily water consumed over 4 h (mL/kg/4 hours) during baseline and during weeks with access to alcohol. Group size = 10. Data are presented as mean ± S.E.M and points represent individual values

Alcohol intake did not differ between the vehicle- and semaglutide-group during the baseline period (F (1, 18) = 2.058, *P* = 0.1686; 3.9 ± 0.22 and 3.6 ± 0.10 for the semaglutide-group and 4.2 ± 0.21 and 3.8 ± 0.13 for the vehicle-group, during the first and second baseline week, respectively). When alcohol was again made available after the up-titration period, the semaglutide-treated animals drank significantly less alcohol relative to vehicle [F (1, 18) = 9.939, *P* = 0.0055], with a significant effect of week [F (2,709, 48,76) = 6.441, *P* = 0.0013] and treatment by week interaction [F (3, 54) = 5.207, *P* = 0.0031] (Fig. 2). Šídák’s multiple comparisons test showed that the semaglutide-treated animals drank significantly less during week 1 (*P* = 0.0428) and 2 (*P* = 0.0022), while the effect did not reach statistical significance in week 3 (*P* = 0.0638). Groups did not differ in alcohol intake during the wash-out period (*P* = 0.9703). Water intake did not differ between the groups [F (1, 18) = 0.6150, *P* = 0.4431]. However, there was a significant effect of week [F (5, 90) = 8.973, *P* < 0.0001] and treatment by week interaction [F (5, 90) = 2.370, *P* = 0.0455] (Fig. 3). Water intake did not differ between the groups during any of the individual weeks (Šídák’s multiple comparisons test). No change in food intake, emetic events or skin irritation at the injection sites were observed. In addition, no significant decline in body weight was observed and the groups did not differ with respect to the amount of body weight gained [F (1, 18) = 0.6165, *P* = 0.4426]. The average weight at start and end of the experiment went from 5.8 kg ± 0.2 kg to 5.9 kg ± 0.2 kg for the semaglutide-group and from 5.5 kg ± 0.2 kg to 5.8 kg ± 0.2 kg for the vehicle-group.

**Fig. 4:**
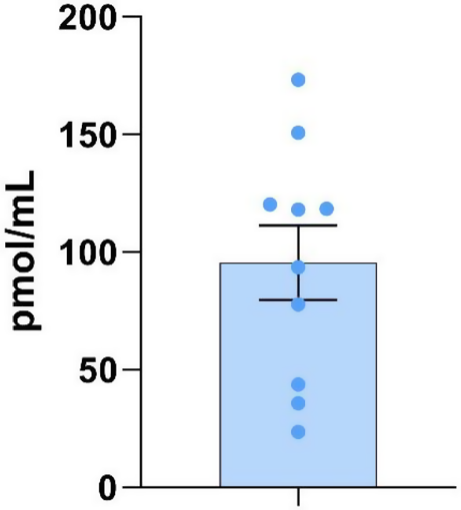
Semaglutide plasma concentration, measured in blood collected in the last week of semaglutide treatment

Average semaglutide plasma concentration, measured in blood collected just after the drinking session on Tuesday in the last week of semaglutide treatment, was 95.5 nM ± 15.8 nM measured in the semaglutide group (Fig. 4). No significant correlation was observed between plasma concentrations of semaglutide and consumption of alcohol during the third treatment week. In the vehicle-group, all values were below the lower limit of quantification.

## Discussion

Recently, several publications have reported on the inhibitory effect of the GLP-1RA semaglutide on alcohol consumption in rodents (Aranäs et al. 2023; Chuong et al. 2023). However, to the best of our knowledge, no studies on its effects on alcohol consumption have been performed in non-human primates. Consequently, we investigated the effect of semaglutide on alcohol intake in alcohol-preferring male vervet monkeys. We demonstrated that the semaglutide group, receiving a dose of 0.05 mg/kg s.c. twice weekly, exhibited a significant reduction in alcohol consumption in comparison to the vehicle-treated control group. During the washout period, the main effect of the treatment on alcohol intake disappeared indicating that a continuous stimulation of GLP-1 receptors is needed to reduce alcohol consumption in alcohol-preferring individuals. The dose used in the main study (0.05 mg/kg s.c. twice weekly) was based on a pilot study in three monkeys. Neither emetic events nor a decrease in food or water consumption or body weight were observed during the dose-finding study or the main study. Our data in vervet monkeys is in accordance with recently published reports on the lowering effect of semaglutide on alcohol intake in rodents (Aranäs et al. 2023; Chuong et al. 2023). Notably, the current findings also extend previous findings from acute to chronic dosing of semaglutide. Previous rodent studies reported repeated acute dosing, whereas the clinically used dosing regimen produces a steady state semaglutide plasma level. Intermittent and continuous dosing can produce diverging effects in terms of tolerance vs. maintained or sensitized effects and tolerability (Lund et al. 2014; Umapathysivam et al. 2014). However, the clinical use of GLP1-RAs in the management of type 2 diabetes has not suggested tolerance to therapeutic effects. In the present study, semaglutide was administered for six weeks in total, and the present data indicates that semaglutide can induce a sustained reduction in alcohol consumption. This finding is in concordance with rodent experiments using repeated administration of exenatide (Thomsen et al. 2017) and liraglutide (Vallöf et al. 2016). However, at week 8 the effect of semaglutide on alcohol intake was at a trend level (Fig. 2). Less profound lowering effects of repeated administration GLP-1 RAs on alcohol intake has also been observed in rodents (Vallof et al., 2016). If would be of interest to further study the effects of repeated administration of semaglutide and other GLP-1 RAs on alcohol intake in rodents, nonhuman primates as well as in humans for a longer time period.

The results support our earlier published data on two GLP-a receptor agonists, exenatide and liraglutide, which, given chronically, both decreased alcohol intake in vervet monkeys (Thomsen et al. 2019, 2020). The effects of semaglutide on alcohol intake were more pronounced than what was observed for exenatide and liraglutide. However, since only a single dose of exenatide, liraglutide and semaglutide was tested, we cannot draw firm conclusions on possible differences in efficacies or potencies between the different GLP-1RAs investigated.

As mentioned earlier, our group has recently studied the effect of the GLP-1RA exenatide on alcohol drinking behaviour and brain activity in patients with AUD who simultaneously received cognitive behavioural therapy. In the exenatide-treated group, alcohol cue-induced brain activation in reward-related areas was significantly lower than in the placebo group. A potent reduction in alcohol intake was observed in both groups with no significant differences between groups. Remarkably, among patients with a BMI exceeding 30, the exenatide-treated group exhibited a noteworthy reduction in both the frequency of heavy drinking days and total alcohol consumption when compared to the placebo group (Klausen et al. 2022a, b). Semaglutide is a more potent GLP-1 receptor agonist than exenatide, and the effect of semaglutide in the present study was also more profound than what we observed in our earlier study with exenatide in alcohol-preferring vervet monkeys (Thomsen et al. 2019). We consequently initiated a new clinical randomized control trial study to study the effect of semaglutide, 2.4 mg subcutaneously, once weekly, on the number of heavy drinking days and total alcohol intake in patients with AUD and a BMI of 30 kg/m^2^ or above (ClinicalTrials.gov identifier: NCT05895643).

No body weight loss was observed in the present study. This is in accordance with unpublished data from our prior study in vervet monkeys investigating the effects of the two GLP-1 RAs exenatide and liraglutide (Thomsen et al. 2019) where no loss in body weight was found (unpublished data). Whether the same holds true in lean humans is an open question. We were not able to find any published data on the effects of semaglutide or other GLP-1 RAs on body weight in lean humans with BMI between 18.5 and 24.9.

We measured the plasma levels of semaglutide at the end of the last week of semaglutide-treatment. The average semaglutide plasma level was at about the same level as the plasma concentration measured in humans (around 80 pmol/mL) following chronic treatment with semaglutide for type 2 diabetes, at a weekly s.c. dose of 2.4 mg (Strathe et al. 2023). We did not find a significant correlation between semaglutide plasma levels and alcohol consumption; this may have been due to the relatively small number of monkeys (*n* = 10) in the semaglutide-treated group. However, in our ongoing randomized, double-blinded, placebo-controlled clinical trial, investigating the effects of semaglutide, 2.4 mg s.c. once weekly on alcohol intake in patients with AUD (ClinicalTrials.gov identifier: NCT05895643), we will investigate a possible correlation between semaglutide plasma levels and consumption of alcohol.

We acknowledge some limitations to the present study. We cannot exclude the possibility that single housing during alcohol testing induces stress. This might influence the test results, as stress is a well-established trigger for relapse in AUD. However, even if our monkeys might have experienced stress, this does not deter from the translational relevance of our data. Also, equally important, both the control group and the semaglutide group were exposed to the same stressor, i.e., individual housing. If this had a prime effect, it would apply equally to both drug and vehicle group. Our earlier data in socially housed mice (Thomsen et al. 2017) did not indicate that this is an issue, but stress-induced alterations in alcohol consumption may differ in non-human primates. Preferably, the monkeys should be tested for alcohol consumption in their group-housed environment. However, this requires sophisticated analytical equipment currently unavailable at the Behavioural Science Foundation research facilities. We have only tested male vervet monkeys; therefore, we cannot conclude that the alcohol intake-lowering effect of semaglutide applies to both sexes. However, relatively few studies have reported on the effects of GLP-1RAs on alcohol seeking and intake in both sexes. Available evidence in rodents suggests some sex-based differences in effect size but generally indicates that GLP-1RAs decreased alcohol intake in both sexes (Aranäs et al. 2023; Chuong et al. 2023; Díaz-Megido and Thomsen 2023; Vallöf et al. 2020). The main reason why female monkeys are not tested in more chronic experimental setups is the potential toxic effect of alcohol on the foetus. Moreover, it would be interesting to test reference compounds used in the clinic against AUD i.e., acamprosate or naltrexone to gauge the relative effect sizes of GLP-1RAs versus reference compounds in the alcohol-preferring vervet monkeys. These experiments on reference compounds are planned.

In conclusion, the present study shows for the first time that the GLP-1RA semaglutide reduces voluntary alcohol drinking in non-human primates without causing emesis, further substantiating the large body of evidence indicating that the GLP-1 receptor could be a possible future target for the treatment of AUD (Klausen et al. 2022a, b; Leggio et al. 2023).
